# Dietary protein and lifespan across the metamorphic boundary: protein-restricted larvae develop into short-lived adults

**DOI:** 10.1038/srep11783

**Published:** 2015-06-29

**Authors:** A. Runagall-McNaull, R. Bonduriansky, A. J. Crean

**Affiliations:** 1 Evolution & Ecology Research Centre, School of Biological, Earth and Environmental Sciences, University of New South Wales, Sydney, 2052 Australia

## Abstract

Restriction of nutrients in the adult diet extends lifespan across a diverse range of species, but less is known about the long-term effects of developmental dietary restriction. In particular, it is not known whether adult lifespan is influenced by developmental caloric restriction or macronutrient balance. We used the nutritional geometry approach to independently manipulate protein and carbohydrate contents of the larval diet in the neriid fly, *Telostylinus angusticollis,* and measured adult lifespan. We found that adult male and female lifespan was shortest when larvae were fed a protein restricted diet. Thus, protein restriction in the larval diet has the opposite effect of protein restriction in the adult diet (which prolongs life in this species and across a wide range of taxa). Adult lifespan was unaffected by larval dietary carbohydrate. These patterns persisted after controlling for larval diet effects on adult body size. We propose that larval and adult protein sources are used for distinct metabolic tasks: during development, dietary protein is used to build a durable soma that enhances adult lifespan, although excessive protein consumption partially reverses this effect.

Moderate restriction of nutrients in the adult diet extends lifespan in species ranging from yeast to primates (reviewed in^1^,^2^). Traditional explanations for lifespan extension effects of adult dietary restriction assume an adaptive allocation of limited resources between two or more metabolic activities^3^, particularly reproductive effort and somatic repair. During periods of famine the likelihood of successful reproduction is small, and therefore an individual may re-allocate energy to somatic maintenance, ostensibly to increase the chance of surviving until conditions improve^4^. This interpretation is challenged by studies showing that lifespan extension is associated with the specific macronutrient composition and balance of the adult diet, rather than overall caloric restriction^5^,^6^,^7^,^8^. For example, an individual forced to feed on a fixed ratio of nutrients may over-consume one nutrient in order to acquire enough of another for a given metabolic task^3^, leading to toxicity effects of the over-consumed nutrient. Higher protein to carbohydrate ratio adult diets are consistently associated with decreased adult lifespan in insects^7^,^9^,^10^, suggesting that the toxic effects of protein overconsumption may be driving the lifespan extension effects of dietary restriction^11^.

Less is known about the effects of developmental diet on adult lifespan. Nutrients acquired during larval stages determine somatic quality and fat reserves, and can therefore influence adult behaviour and life history strategies^3^,^12^,^13^. Restriction of nutrients in larval stages is generally considered to negatively influence adult fitness by increasing development time and reducing adult size, male secondary sexual trait expression, and both male and female fecundity^13^,^14^,^15^,^16^. However, effects of larval dietary restriction on adult lifespan are inconsistent, with studies finding no effect^14^,^15^,^17^, positive effects^18^, negative effects^13^, and sex-specific effects^12^,^19^,^20^. Because none of these studies manipulated macronutrient availability independently of macronutrient ratio, it is not known whether these effects are caused by caloric restriction, protein restriction, or variation in nutrient balance.

We used a nutritional geometry approach^21^ to examine the separate and interactive effects of protein and carbohydrate in the larval diet on adult lifespan of the neriid fly *Telostylinus angusticollis* (Fig. 1). Previous studies in this species have shown that restriction of adult dietary protein increases lifespan by 67%^19^. In addition, previous studies have shown that larvae reared on a nutrient-poor medium develop into smaller adults^16^ that are more susceptible to starvation^19^. Increased concentrations of carbohydrates in the larval diet increases egg-to-adult viability and adult body size, whereas protein in the larval diet enhances the expression of male secondary sexual traits but decreases larval survival^22^. The effects of protein and carbohydrate consumption by larvae on adult lifespan have not been examined previously. Adults were provided with both protein and sugar to test for effects of developmental dietary restriction independently of adult dietary restriction, and focal individuals were housed with a standardised individual of the opposite sex to allow for reproduction.

## Results

There was no significant effect of larval dietary protein or carbohydrate on egg-to-adult viability in the range of concentrations tested (Table 1a). Adult body size was positively influenced by both protein and carbohydrate concentrations in the larval diet, positively related to the number of adults that emerged per replicate, and negatively related to development time (Table 1b). Accounting for this effect on adult body size, adult lifespan was related to protein, but not related to carbohydrate concentrations in the larval diet, although there was a borderline significant negative protein × carbohydrate interaction effect (Table 1c). Larval dietary protein had a positive effect on adult lifespan up to intermediate levels, with flies reared on 11 g/L protein concentration diets living 73% longer on average than flies reared on protein concentrations below 3 g/L (mean lifespan [days ± s.e.]: protein 2.7 g/L = 56.53 ± 6.74; protein 11 g/L = 97.74 ± 2.83; Fig. 2). However, very high protein concentrations (above 30 g/L) resulted in decreased lifespan, reflected in a negative quadratic protein effect (Table 1c), particularly when diets also contained high concentrations of carbohydrates (Fig. 2). There was no significant effect of sex, body size, development time, or number of adults emerged per replicate on adult lifespan (Table 1c). Removing body size from the model did not qualitatively change the effects of larval dietary protein or carbohydrate on adult lifespan.

## Discussion

*Telostylinus angusticollis* larvae reared on protein-restricted diets suffered reduced adult lifespan. This negative effect of protein restriction in the larval diet contrasts starkly with the well-known lifespan extension effects of protein restriction in the adult diet^1^,^2^. Minimizing dietary protein in adult stages maximises lifespan in *T. angusticollis*^19^, *Drosophila*^7^, the Queensland fruit fly *Bactocera tryoni*^10^, and the field cricket *Teleogryllus commodus*^9^. In contrast, we found that protein in the larval diet had a positive impact on adult lifespan up to intermediate levels (approx. 20 g/L). This positive effect of larval dietary protein (a 73% increase in lifespan between low and intermediate protein concentrations) is comparable in magnitude to the negative effect of adult dietary protein in this species (adult flies fed a protein-restricted diet live 67% longer^19^). However, further increases in dietary protein at the larval stage reduced adult lifespan, consistent with toxic effects of high protein concentrations in the larval diet previously demonstrated in *T. angusticollis*^22^. By using the powerful geometric framework, we were able to decouple effects of protein from effects of carbohydrates. The non-linear effects of protein concentration and non-significant effect of carbohydrate concentration found in this study may explain the inconsistent results of previous studies examining effects of larval diet composition on adult lifespan^19^.

Flies were fed *ad libitum* after adult emergence to allow for reproduction, as adult dietary restriction can render females completely infertile (ovaries fail to develop)^19^. Our results suggest that flies were unable to use protein in the adult diet to compensate for developmental protein restriction. Hence, it appears that protein consumed during each lifecycle stage is being used for different metabolic tasks. However, we are unable to assess interactive effects between the larval and adult diet as adult diet manipulations were not included in the study design for logistic reasons. In holometabolous insects the larval stage represents the only opportunity for investment in the growth of the adult soma and as such, any contribution that soma quality makes to lifespan ought to reflect the level of nutrition available during development. In contrast, protein in the adult stage is used primarily in reproduction, and *T. angusticollis* females deprived of protein as adults are unable to produce eggs^19^. The distinct uses for dietary protein between life-history stages may explain why protein has opposite effects on lifespan in developmental versus adult diets. Dietary protein contains the necessary raw materials for development^23^ and promotes the release of insulin-like growth factors (IGF) that stimulate cellular growth and proliferation in *Drosophila*^24^. Protein consumed during development may therefore result in a durable or resilient soma, which in turn contributes positively to lifespan. Further studies are required to determine whether investment in musculature, exoskeleton quality or some other somatic component is responsible for the lifespan extension associated with protein rich larval diets. Further research, quantifying effects on adult reproductive output, is also required to determine how larval nutrition affects net fitness.

Surprisingly, the only effect of larval carbohydrate consumption on adult lifespan detected was a borderline significant negative protein × carbohydrate interaction effect, even though carbohydrates enhanced egg-to-adult viability and adult body size. Theoretical work by Boggs^3^,^25^ suggests that resource intake is not necessarily proportional to expenditure at any one lifecycle stage, and previous studies show larval diet quality can contribute to adult resource reserves^13^,^26^,^27^. However, fat reserves may only be important for early adult survival and reproduction^28^, and flies may be able to use carbohydrates (but not protein) in the adult diet to compensate for larval dietary restriction. We also found no relationship between adult lifespan and replicate viability or development time, suggesting that the effect of larval protein on adult lifespan was not associated with selection for higher quality individuals in replicates with lower emergence, nor mediated by protein effects on development time^14^.

Protein consumption during development may have had a different effect on observed lifespan had adults (particularly males) been maintained in a different social environment (e.g. in isolation, or in a highly competitive environment, rather than in male-female pairs). Depending on their condition and perceived probability of mating success, males may sacrifice potential lifespan for increased reproductive success (reviewed in^29^). For example, large male antler flies mate more frequently in early life but undergo rapid reproductive ageing^30^, whilst high condition male field crickets invest more heavily in costly sexual display (calling) than low condition males, resulting in body mass loss and decreased lifespan^12^. *T. angusticollis* males reared on protein-rich larval diets exhibit enhanced secondary sexual trait expression^22^. Hence, it is possible that the effects of larval dietary protein on adult male lifespan may be reversed within a competitive adult environment. Similarly, all flies were housed with a standardized individual of the opposite sex to allow for reproduction. However, as treatment individuals varied in size, the relative size of the treatment individual to the standardized mate varied across treatments, potentially affecting mating rates. Further work is necessary to fully understand the relative contributions of larval and adult environment to lifespan. Nevertheless, it is clear that both developmental and adult diets can affect adult lifespan, and our results suggest that protein ingestion before and after metamorphosis can have very different effects on adults.

## Methods

### Study species

*Telostylinus angusticollis* (Diptera, Neriidae) displays pronounced condition-dependent sexual dimorphism in response to larval diet^16^. Males reared on a nutrient-rich medium are much larger and have enhanced secondary sexual traits^16^,^22^, and adults of both sexes may have a competitive advantage over those reared under restricted larval nutrients^31^. In the wild, individuals aggregate and oviposit on decaying tree bark. Wild flies of both sexes exhibit far higher extrinsic (age-independent) mortality rates and much shorter life spans than flies reared in the laboratory^32^. Mean lifespan in the laboratory ranges from 20 to 65 days, depending on diet and social environment^19^,^33^. Protein restriction in the adult diet extends lifespan of both sexes, but the costs on fecundity are severe for females and subtle for males^19^.

Eggs for this experiment were taken from a lab stock originating from Fred Hollows Reserve, Sydney, Australia (recently supplemented with new wild-caught flies from the same source), and reared on a standardised larval diet (‘rich’- see ref [16]) for one generation prior to the experimental diet manipulation to minimize any parental effects associated with ancestral diets.

### Treatment diets

**Figure 1:**
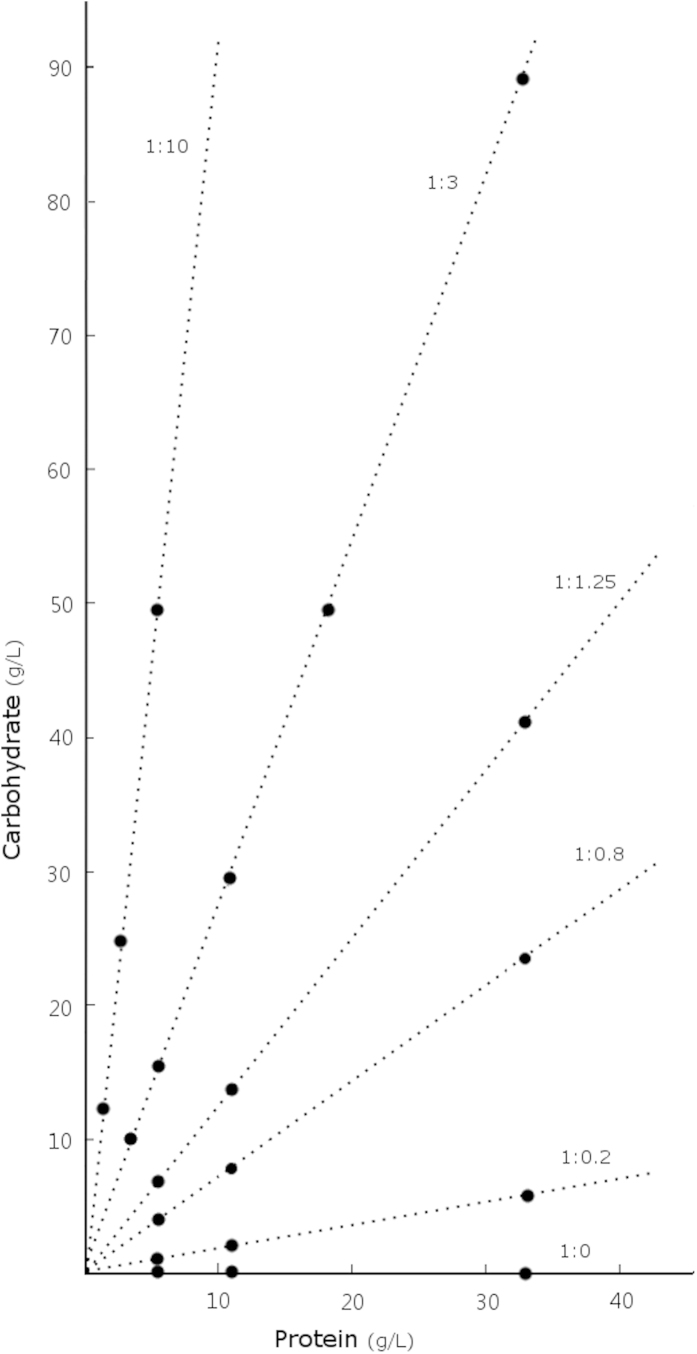
Nutrient space with six rails representing each P:C ratio used in this experiment. Points on each rail represent treatment diets with varying quantities of protein and carbohydrate (g) per 1 L of dry cocopeat and 600 mL water.

**Figure 2:**
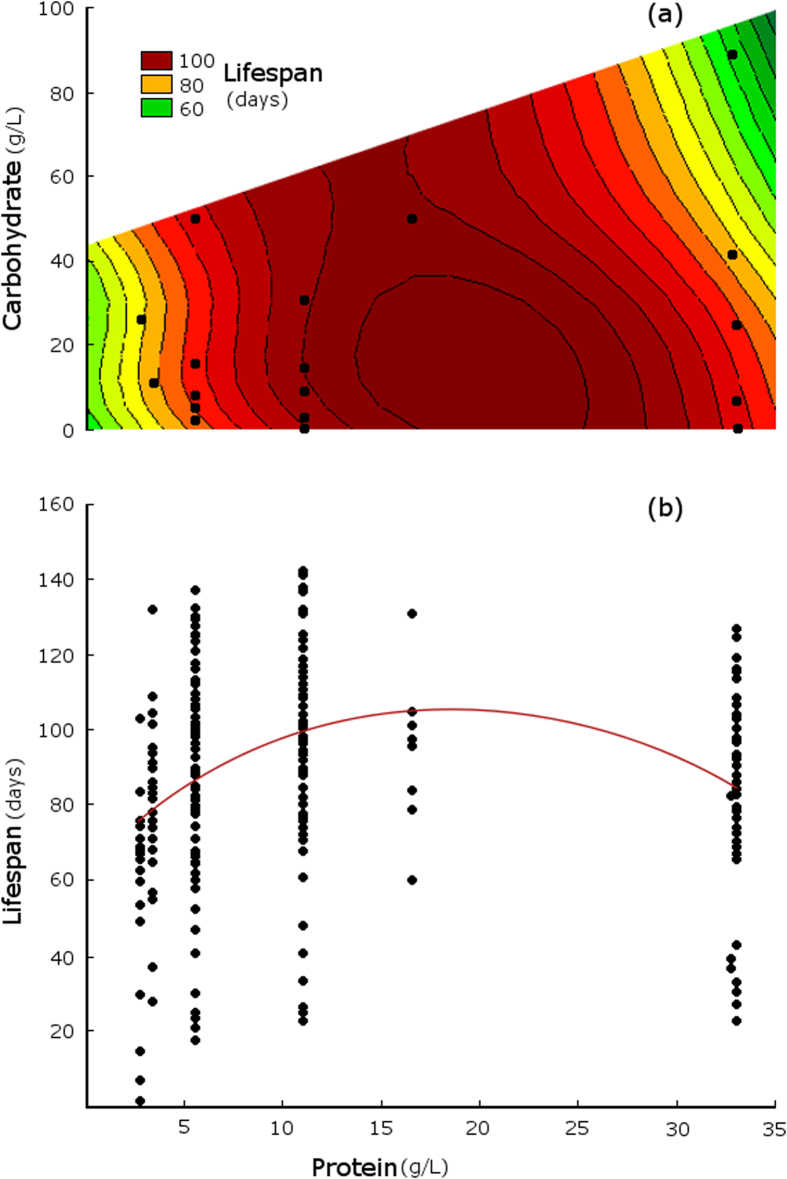
Effect of larval nutrients on adult lifespan. (**a**) Response surface for adult lifespan as a function of protein and carbohydrate content of the larval diet. Values of the response variable (lifespan in days) are indicated by colour, based on thin-plate spline projection fitted to replicate means. Points show larval diets. (**b**) Adult lifespan as a function of protein content (g/L) in the larval diet. Points show individual data points, line shows quadratic fit.

Twenty treatment diets consisted of varying quantities of soy protein (Nature’s Way brand; Pharm-a-care Pty. Ltd., Warriewood, NSW, Australia) and brown sugar, added to 1 L of dry cocopeat (Galuku Pty. Ltd., Sydney, NSW, Australia), hydrated with 600 ml of water and homogenised thoroughly with a handheld electric mixer (see Supplementary Table 1). The brown sugar used in these experimental diets consists of 98% (by weight) fructose, sucrose and other sugars, minerals (primarily sodium) and 0.2% protein. The soy protein used contains 18 amino acids (Alanine, Arginine, Aspartic Acid, Cysteine, Glutamic Acid, Glycine, Histidine, Isoleucine, Leucine, Lysine, Methionine, Phenylalanine, Proline, Serine, Threonine, Tryptophan, Tyrosine, Valine); and the cocopeat contains only negligible levels of available nutrients for developing fly larvae. The 20 larval diets represent 6 P:C ratios with multiple concentrations of each ratio (Fig. 1), adapted from^22^. High-protein diets associated with very low emergence were replaced with other diets in the present study.

For each larval diet, we set up 5 replicate 250 ml containers of ~100 g larval medium, sufficient to be considered *ad libitum* for 20 larvae^31^. Twenty eggs were transferred to each larval container (20 diets × 5 containers = 100 replicate containers in total). Clutches of eggs laid by a single female were divided amongst several replicate containers, randomly alternating among treatments to control for genetic variation, parental age, and environment. The replicate containers were maintained at 27 °C and 50% humidity in an environment chamber, and moistened with water every three days. After 21 days, when most larvae had pupated, replicate containers were removed from the environment chamber and each placed into a 2 L container to record adult emergence (Supplementary Table 2). Each 2 L container included a layer of moist cocopeat on the bottom and *ad libitum* brown sugar and yeast. The first three male and female F1 adults to emerge from each replicate were removed, housed individually in a 250 ml cage with a substrate of moist cocopeat and provided with *ad libitum* brown sugar and yeast. Containers were checked and watered daily. After 7 days an individual of the opposite sex (raised on the standard ‘rich’ larval diet^16^) was added to each individual container and after 14 days a Petri dish of oviposition medium was provided to allow oviposition to occur. The oviposition medium was removed after 14 days. Pairs were maintained at 27 °C on a 14:10 day/night cycle until the death of the focal individual in each pair, which was then frozen at −20 °C for measurement of body size (thorax length). Non-focal individuals were removed upon death, but were not replaced. We ended the experiment whilst six focal individuals were still alive, and thus lifespan was capped at 142 days.

### Analysis

The effects of larval dietary protein (P), carbohydrate (C), their quadratic terms (P^2^, C^2^), and cross-product (P × C) on egg-to-adult viability were analysed using a generalized linear model with a ‘quasi-Poisson’ correction for over-dispersion. Data on individual adult body size (thorax length standardized within sex) and adult lifespan (days from adult emergence until death) were analysed using general linear mixed models (REML), with amount of larval protein (P) and carbohydrate (C) and their quadratic terms (P^2^, C^2^) and cross-product (P × C), replicate viability (number of adults emerged per replicate), development time (days from egg to emergence), and Replicate (random factor) included as predictor variables. Sex and standardized adult body size were also included as covariates in the model of adult lifespan. As not all replicate containers produced viable adults, the number of replicates contributing to body size and lifespan analyses was reduced to 75 (see Supplementary Table 2). Random distribution of model residuals was verified by inspection. Effects of larval dietary components on adult lifespan were visualised using a thin-plate spline projection in two dimensions (protein and carbohydrate), with colour representing the response dimension. All data were analysed using JMP (version 10.0.0, SAS Institiute).

## Additional Information

**How to cite this article**: Runagall-McNaull, A. *et al.* Dietary protein and lifespan across the metamorphic boundary: protein-restricted larvae develop into short-lived adults. *Sci. Rep.* **5**, 11783; doi: 10.1038/srep11783 (2015).

## Supplementary Material

Supplementary Information

## Figures and Tables

**Table 1:** Effects of larval dietary protein and carbohydrate and their squares and product on (a) number of adults emerged per replicate (egg-to-adult viability); **(b)** adult body size (standardized within sex), and **(c)** adult lifespan (days from emergence to death). Significant coefficients are highlighted in bold.

	**(a) Egg-to-adult viability**			**(b) Adult body size**			**(c) Adult lifespan**		
	**Estimate**	**s.e.**	**p**	**Estimate**	**s.e.**	**p**	**Estimate**	**s.e.**	**p**
Protein	0.130	0.084	0.115	**0.223**	0.031	<0.001	**3.211**	0.913	0.001
Carbohydrate	0.072	0.041	0.056	**0.047**	0.008	<0.001	0.295	0.264	0.266
Protein^2^	−0.004	0.002	0.057	**−0.005**	0.001	<0.001	**−0.079**	0.022	0.001
Carbohydrate^2^	−0.001	0.001	0.093	<0.001	<0.001	0.846	−0.001	0.004	0.753
Protein × Carbohydrate	−0.001	0.002	0.458	−0.001	<0.001	0.054	−0.018	0.009	0.052
development time				**−0.048**	0.010	<0.001	−0.315	0.477	0.511
replicate viability				**0.069**	0.013	<0.001	0.385	0.394	0.331
sex							−1.511	1.416	0.287
body size							3.931	2.524	0.122
Replicate (random factor) % of residual variance:				59.079%			<0.001%		
