# Supplementary material for: Dietary protein and lifespan across the metamorphic boundary: protein-restricted larvae develop into short-lived adults
Source: Sci Rep. 2015 Jun 29;5:11783. doi: 10.1038/srep11783 (PMC4484247; doi:10.1038/srep11783)
Supplement: Supplementary Information [file srep11783-s1.doc]

**Dietary protein and lifespan across the metamorphic boundary: protein-restricted larvae develop into short-lived adults**

A. Runagall-McNaull, R. Bonduriansky and A. J. Crean*

Evolution & Ecology Research Centre

School of Biological, Earth and Environmental Sciences

University of New South Wales

Sydney, 2052 Australia

*Communicating author: a.crean@unsw.edu.au

**Supplementary Table 1: Amount (g) of soy protein and brown sugar used to prepare each of the 20 experimental diets. Quantities are per 1L dry cocopeat and 600mL water.**

|  | **P:C Ratio** | | | | | |
| --- | --- | --- | --- | --- | --- | --- |
|  | **1:0** | **1:0.2** | **1:0.8** | **1:1.4** | **1:3** | **1:10** |
| **Nutrient** | **Amount (g)** | | | | | |
| **Protein** | 33 | 33 | 33 | 32.9 | 32.8 |  |
| **Sugar** | 0 | 5.9 | 23.7 | 41.3 | 89 |  |
|  |  |  |  |  |  |  |
| **Protein** |  |  |  |  | 16.5 |  |
| **Sugar** |  |  |  |  | 49.4 |  |
|  |  |  |  |  |  |  |
| **Protein** | 11 | 11 | 11.1 | 11 | 10.9 |  |
| **Sugar** | 0 | 2 | 7.9 | 13.8 | 29.7 |  |
|  |  |  |  |  |  |  |
| **Protein** | 5.5 | 5.5 | 5.5 | 5.5 | 5.5 | 5.4 |
| **Sugar** | 0 | 1 | 4 | 6.9 | 14.8 | 49.4 |
|  |  |  |  |  |  |  |
| **Protein** |  |  |  |  | 3.3 | 2.7 |
| **Sugar** |  |  |  |  | 10 | 24.7 |
|  |  |  |  |  |  |  |
| **Protein** |  |  |  |  |  | 1.35 |
| **Sugar** |  |  |  |  |  | 12.35 |

**Supplementary Table 2:** Number of individuals emerged per replicate and sample sizes used in the lifespan assay.

| P:C Ratio | Protein (g/L) | # adults emerged | # females in lifespan assay | # males in lifespan assay |
| --- | --- | --- | --- | --- |
| 1 to 0 | 33 | 5 | 3 | 1 |
| 1 to 0 | 33 |  | 3 | 3 |
| 1 to 0 | 33 | 6 | 2 | 3 |
| 1 to 0 | 33 | 2 | 2 |  |
| 1 to 0 | 33 | 7 | 3 | 1 |
| 1 to 0 | 11 | 0 |  |  |
| 1 to 0 | 11 | 0 |  |  |
| 1 to 0 | 11 | 1 | 1 |  |
| 1 to 0 | 11 | 0 |  |  |
| 1 to 0 | 11 | 1 | 1 |  |
| 1 to 0 | 5.5 | 0 |  |  |
| 1 to 0 | 5.5 | 0 |  |  |
| 1 to 0 | 5.5 | 0 |  |  |
| 1 to 0 | 5.5 | 0 |  |  |
| 1 to 0 | 5.5 | 0 |  |  |
| 1 to 0.2 | 33 | 0 |  |  |
| 1 to 0.2 | 33 | 3 | 2 | 1 |
| 1 to 0.2 | 33 | 5 | 3 | 2 |
| 1 to 0.2 | 33 | 1 |  | 1 |
| 1 to 0.2 | 33 | 0 |  |  |
| 1 to 0.2 | 11 | 3 | 1 | 2 |
| 1 to 0.2 | 11 | 6 | 3 | 3 |
| 1 to 0.2 | 11 | 8 |  | 3 |
| 1 to 0.2 | 11 | 12 | 3 | 3 |
| 1 to 0.2 | 11 | 8 | 3 | 1 |
| 1 to 0.2 | 5.5 | 0 |  |  |
| 1 to 0.2 | 5.5 | 0 |  |  |
| 1 to 0.2 | 5.5 | 0 |  |  |
| 1 to 0.2 | 5.5 | 0 |  |  |
| 1 to 0.2 | 5.5 | 1 |  | 1 |
| 1 to 0.8 | 33 | 1 |  | 1 |
| 1 to 0.8 | 33 | 0 |  |  |
| 1 to 0.8 | 33 | 3 | 2 | 1 |
| 1 to 0.8 | 33 | 7 | 3 | 2 |
| 1 to 0.8 | 33 | 2 | 1 | 1 |
| 1 to 0.8 | 11 | 17 | 3 | 3 |
| 1 to 0.8 | 11 | 15 | 3 | 3 |
| 1 to 0.8 | 11 | 19 | 3 | 3 |
| 1 to 0.8 | 11 | 13 | 3 | 3 |
| 1 to 0.8 | 11 | 19 | 3 | 3 |
| 1 to 0.8 | 5.5 | 16 | 3 | 3 |
| 1 to 0.8 | 5.5 | 13 | 3 | 3 |
| 1 to 0.8 | 5.5 | 16 | 3 | 3 |
| 1 to 0.8 | 5.5 | 17 | 3 | 3 |
| 1 to 0.8 | 5.5 | 14 | 3 | 3 |
| 1 to 1.4 | 32.9 | 0 |  |  |
| 1 to 1.4 | 32.9 | 1 |  | 1 |
| 1 to 1.4 | 32.9 | 5 | 3 | 1 |
| 1 to 1.4 | 32.9 | 2 | 2 |  |
| 1 to 1.4 | 32.9 | 0 |  |  |
| 1 to 1.4 | 11 | 16 | 3 | 2 |
| 1 to 1.4 | 11 | 9 | 3 | 2 |
| 1 to 1.4 | 11 | 17 | 3 | 2 |
| 1 to 1.4 | 11 | 16 | 3 | 3 |
| 1 to 1.4 | 11 | 18 | 3 | 3 |
| 1 to 1.4 | 5.5 | 15 | 3 | 3 |
| 1 to 1.4 | 5.5 | 16 | 2 | 3 |
| 1 to 1.4 | 5.5 | 18 | 3 | 3 |
| 1 to 1.4 | 5.5 | 18 | 3 | 3 |
| 1 to 1.4 | 5.5 | 18 | 3 | 3 |
| 1 to 3 | 32.8 | 2 |  | 2 |
| 1 to 3 | 32.8 | 0 |  |  |
| 1 to 3 | 32.8 | 1 | 1 |  |
| 1 to 3 | 32.8 | 1 |  | 1 |
| 1 to 3 | 32.8 | 0 |  |  |
| 1 to 3 | 16.5 | 1 | 1 |  |
| 1 to 3 | 16.5 | 0 |  |  |
| 1 to 3 | 16.5 | 1 | 1 |  |
| 1 to 3 | 16.5 | 4 |  | 3 |
| 1 to 3 | 16.5 | 5 | 3 | 1 |
| 1 to 3 | 10.9 | 9 | 3 | 3 |
| 1 to 3 | 10.9 | 17 | 3 | 3 |
| 1 to 3 | 10.9 | 8 | 3 | 3 |
| 1 to 3 | 10.9 | 16 | 3 | 3 |
| 1 to 3 | 10.9 | 15 | 3 | 2 |
| 1 to 3 | 5.5 | 19 | 3 | 3 |
| 1 to 3 | 5.5 | 20 | 3 | 2 |
| 1 to 3 | 5.5 | 17 | 3 | 3 |
| 1 to 3 | 5.5 | 19 | 3 | 3 |
| 1 to 3 | 5.5 | 19 | 3 | 3 |
| 1 to 3 | 3.3 | 10 | 3 | 2 |
| 1 to 3 | 3.3 | 11 | 3 | 3 |
| 1 to 3 | 3.3 | 9 | 3 | 3 |
| 1 to 3 | 3.3 | 13 | 3 | 3 |
| 1 to 3 | 3.3 | 6 | 3 | 2 |
| 1 to 10 | 5.4 | 12 | 3 | 3 |
| 1 to 10 | 5.4 | 9 | 3 | 3 |
| 1 to 10 | 5.4 | 10 | 3 | 3 |
| 1 to 10 | 5.4 | 12 | 2 | 3 |
| 1 to 10 | 5.4 | 7 | 3 | 3 |
| 1 to 10 | 2.7 | 14 | 3 | 3 |
| 1 to 10 | 2.7 | 6 | 1 | 3 |
| 1 to 10 | 2.7 | 1 | 1 |  |
| 1 to 10 | 2.7 | 1 | 1 |  |
| 1 to 10 | 2.7 | 7 | 2 | 3 |
| 1 to 10 | 1.35 | 0 |  |  |
| 1 to 10 | 1.35 | 0 |  |  |
| 1 to 10 | 1.35 | 0 |  |  |
| 1 to 10 | 1.35 | 0 |  |  |
| 1 to 10 | 1.35 | 0 |  |  |
